# Trimethylamine *N*-Oxide and mortality in hemodialysis patients: like a mystery

**DOI:** 10.1080/0886022X.2021.1890618

**Published:** 2021-03-03

**Authors:** Yu Zhao, Wenyun Wang, Zhilong Dong

**Affiliations:** a Department of Medicine, Northwest University for Nationalities, Lanzhou, PR China; b Department of Pediatric Surgery, Second Hospital of Lanzhou University, Lanzhou, PR China; c Department of Urology, Second Hospital of Lanzhou University, Lanzhou, PR China

Dear Sir,

I have read with great interest the paper entitled ‘Association of trimethylamine *N*-Oxide with cardiovascular and all-cause mortality in hemodialysis patients’ by Zhang et al. [1]. Trimethylamine *N*-Oxide (TMAO) is a proatherogenic and prothrombotic metabolite. The article recruited 252 hemodialysis (HD) patients from China with a mean TMAO of 4.73 µg/ml (62.9 μmol/l) and divided them into a high-TMAO group (>4.73 µg/ml) and a low-TMAO group (≤4.73 µg/ml). TMAO was measured by liquid chromatography/mass spectrometry using TMAO-D9 as the internal standard. It found that a higher incidence of cardiovascular death (Log-Rank: *p* = 0.006) and all-cause death (Log-Rank: *p* < 0.001) in the high-TMAO group by Kaplan–Meier analysis. The multivariate Cox regression analysis showed that high TMAO levels were significantly associated with cardiovascular and all-cause mortality in models 1–8, which are displayed in Table 3 and Table 4.

Trimethylamine *N*-Oxide (TMAO) is an enterogenous micromolecular toxin that is mainly synthesized by the gut microflora, and which is filtered by the kidney and excreted in the urine. Plasma TMAO levels in HD patients reflect the net effect of TMAO production and clearance. HD clears TMAO at a rate similar to creatinine, with re-accumulation to plasma concentrations of approximately 100uM before the subsequent dialysis session [2]. However, the present study by Zhang et al. [1]. is that TMAO was measured only once at predialysis, but also does not pay attention to the influence of diet. Although the findings that patients with CKD who have elevated TMAO levels have poor cardiovascular outcomes [3], which extend to patients undergoing HD, I pay special attention to the results of this research because it caused me some confusion.

Firstly, Kaysen et al. [4]. performed a prospective, multicenter cohort study in 235 HD patients with 62 ± 14 years from the Comprehensive Dialysis Study (CDS) to investigate the relationship of TMAO with cardiovascular or all-cause mortality. The median TMAO concentration was 43 μmol/l (25th–75th percentile 28-67uM/l) by using a Waters Acuity UPLC in tandem with an ABSciex 5600Teiple TOF mass spectrometer, and were divided into four group based on TMAO levels (Q1 ≤ 27.5, Q2 27.5–43.0, Q3 43.0–66.6, Q4 66.6–184). Cox model analysis showed that higher serum concentrations (>27.5 μmol/l) of TMAO was no significantly associated with time to death (HR 0.85, 95% CI: 0.65–1.09, *p* = 0.19) or time to cardiovascular hospitalization or cardiovascular death (HR 0.88, 95% CI: 0.57–1.35, *p* = 0.55). Two independent, nested case-control studies conducted by Kalim et al. [5] also did not find a significant association between TMAO and cardiovascular mortality (OR 0.9, 95% CI: 0.7–1.1, *p* = 0.36), assessed by claim data, using a case-control design in the incident HD Accelerated Mortality on Renal Replacement cohort.

Secondly, Stubbs et al. [6]. examined samples from 1243 HD patients in the control arm of the Evaluation of Cinacalcet Hydrochloride Therapy to Lower Cardiovascular Events trial and reported that cardiovascular or all-cause mortality was no association with serum TMAO. Approximately 80% of participants exhibiting TMAO concentrations were ≥56 μmol/l and a maximum TMAO concentration was1103.1 μmol/l by using ultrahigh-performance liquid chromatography-tandem mass spectrometry using heated electrospray ionization. The study cohort was divided into five groups according to baseline TMAO level (Q1 ≤ 56.6, Q2 56.7–79.5, Q3 79.6–107.8, Q4 107.9–155.2, Q5 > 155.2). Cox proportional hazards models analysis showed that no significant difference was found in cardiovascular mortality (HR 1.05, 95% CI: 0.85–1.29, *p* > 0.05) and all-cause mortality (HR 0.94, 95% CI: 0.80–1.10, *p* > 0.05). An additional analysis evaluating the association between TMAO quintiles and cardiovascular outcome yielded similar findings to our models utilizing ln (TMAO).

Finally, Shafi et al. [7]. included 1232 participants (white 431 and black 801) from the HEMO Study to determine the relationship of TMAO with cardiovascular outcomes in HD patients. Mean TMAO concentrations were similar between whites (98 ± 57 μmol/l) and blacks (104 ± 67 μmol/l, *p* = 0.15) and were measured by liquid chromatography/mass spectrometry using TMAO-D9 as the internal standard. In whites, 2-fold higher TMAO was associated with a higher risk of cardiac death, sudden cardiac death, and any-cause death. However, in blacks, the association was nonlinear and significant only for cardiac death among patients with TMAO concentration below the median(88 μmol/l) (HR 1.58, 95% CI: 1.03–2.44, *p* = 0.04). Above the median, there was no association with cardiac death.

In conclusion, I think the inconsistency of study results may partly be associated with measure methods, dialysis modes, residual renal function, intestinal microbiota, diet and the accurately defined difference between high and low levels. Further prospective studies of larger sample size and multiple centers and multiple races and multiple times to record TMAO may help to confirm the relationship and to clarify the potential utility of clinical interventions that target TMAO. Maybe TMAO difference between pre-dialysis and post-dialysis is the best indicator to evaluate the relationship.
